# Efficient network disintegration under incomplete information: the comic effect of link prediction

**DOI:** 10.1038/srep22916

**Published:** 2016-03-10

**Authors:** Suo-Yi Tan, Jun Wu, Linyuan Lü, Meng-Jun Li, Xin Lu

**Affiliations:** 1 College of Information System and Management, National University of Defense Technology, Changsha, Hunan, 410073, P. R. China; 2 Alibaba Research Center for Complexity Sciences, Alibaba Business College, Hangzhou Normal University, Hangzhou, Zhejiang, 311121, P. R.China; 3 Big Data Research Center, University of Electronic Science and Technology of China, Chengdu 611731, China; 4 Department of Public Health Sciences, Karolinska Institutet, Stockholm, 17177, Sweden

## Abstract

The study of network disintegration has attracted much attention due to its wide applications, including suppressing the epidemic spreading, destabilizing terrorist network, preventing financial contagion, controlling the rumor diffusion and perturbing cancer networks. The crux of this matter is to find the critical nodes whose removal will lead to network collapse. This paper studies the disintegration of networks with incomplete link information. An effective method is proposed to find the critical nodes by the assistance of link prediction techniques. Extensive experiments in both synthetic and real networks suggest that, by using link prediction method to recover partial missing links in advance, the method can largely improve the network disintegration performance. Besides, to our surprise, we find that when the size of missing information is relatively small, our method even outperforms than the results based on complete information. We refer to this phenomenon as the “comic effect” of link prediction, which means that the network is reshaped through the addition of some links that identified by link prediction algorithms, and the reshaped network is like an exaggerated but characteristic comic of the original one, where the important parts are emphasized.

Complex networks describe a wide range of systems in nature and society^1^,^2^,^3^. Examples include the Internet, metabolic networks, electric power grids, supply chains, urban road networks, and the world trade web among many others. The study of complex networks has become an important area of multidisciplinary research involving physics, mathematics, biology, social sciences, informatics, and other theoretical and applied sciences. Due to its broad applications, research on the structural robustness of complex networks, i.e., the ability to endure threats and survive accidental events, has received growing attention^4^,^5^,^6^,^7^,^8^,^9^ and has become one of the central topics in the complex network research.

In the majority of cases, networks are beneficial, such as power grids and Internet, where we want to preserve their function. Many studies have considered methods for maximizing the structural robustness of these beneficial networks^10^,^11^,^12^,^13^,^14^,^15^,^16^. In another situation by which this paper is motivated, however, we want to disintegrate a network if it is harmful, such as immunizing a population in social networks or suppressing the virus propagation in computer networks. The immunization problem is mathematically equivalent to asking how to disintegrate a given network with a minimum number of node removals^17^, which is very important since in most cases the number of immunization doses is limited or very expensive. Other examples of network disintegration include destabilizing terrorist networks^18^, preventing financial contagion^19^, controlling the rumor diffusion^20^, and perturbing cancer networks^21^. Although the problem of network disintegration attracts less attention than the problem of network protection, some related works have been devoted to the study of the disintegration strategy. For example, Holme *et al.*^22^ compared the effect of four different targeted disintegration strategies: high degree and betweeness centrality, and their corresponding adaptive versions where the degree (betwenness) of the remaining node is recomputed after each node removal. They found that the removals by the two adaptive methods outperform the two original static methods. Chen *et al.*^23^ developed a new immunization strategy, called the “equal graph partitioning” (EGP) strategy. The main idea of the EGP method is to fragment the network into many connected clusters of equal size, which requires 5% to 50% fewer immunization doses compared to the classical targeted strategy. Schneider *et al.*^24^ developed an immunization approach based on optimizing the susceptible size, which outperforms the best known strategy based on immunizing the highest-betweenness links or nodes.

In the early works on network disintegration, it was usually assumed that the attacker can obtain perfect information on the network structure, in other words, they assumed that the observed networks are complete. However, the complete information of network structure is not always available in realistic cases. Growing attention has been paid to the study of network disintegration with imperfect information. Dezső *et al.*^25^ proposed a biased treatment strategy against viruses spreading based on uncertain information, in which the likelihood of identifying and administering a cure to an infected node depends on its degrees as *k*^*α*^. Li *et al.*^26^ studied the optimal attack problem based on incomplete information, which means that one can obtain the information of partial nodes, when the information is certain. Moreover, many researches^27^,^28^,^29^,^30^ focused on the disintegration strategy based on local information, i.e. the knowledge of the neighborhood.

Different from the above studies which consider either uncertain information or partial information of individual level, in this paper we focus on another important and frequent scenario of imperfect information, such that part of links (i.e., interactions between nodes) are missing in the observed network. In many real networks, such as food webs^31^, terrorist networks^32^, sexual contact networks^33^, protein-protein interaction networks^34^, and disease relationship networks^35^, it is easy to obtain the information of nodes, but difficult to detect the relations or interactions between nodes, which is usually costly or even infeasible. The missing links may reduce the network disintegration performance. To address this problem, a potential approach is to recover the missing links (or part of the missing links), which remind us the so-called “link prediction” problem^36^. Link prediction algorithms aim at estimating the likelihood of the existence of a link between two nodes based on the observed network structure and the attributes of nodes. Therefore, before the attack we can use one of the link prediction algorithms to recover parts of the missing links and then identify the targets based on the “improved” network. Experiments on both synthetic and real networks show that with the assistance of link prediction the performance of disintegration can be largely improved.

## Results

### Network disintegration model based on link prediction

**Figure 1:**
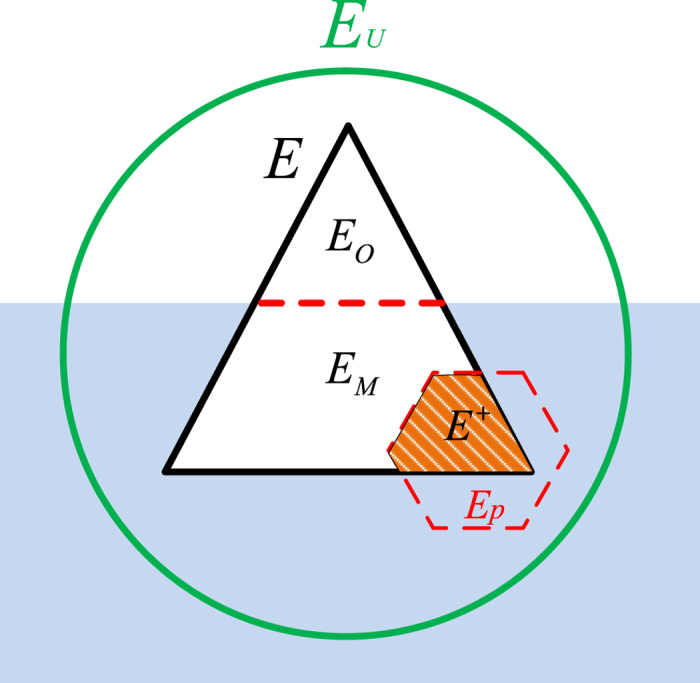
Iceberg diagram for link prediction problem. The triangle represents the set of links *E*, i.e., the complete information, which is divided into two parts: above the sea level is the observed part *E*_*O*_, below the sea level is the invisible (missing) part *E*_*M*_. The hexagon represents the set of predicted links, namely *E*_*P*_. The polygon filled by stripes represents the set of links that are predicted right, namely *E*^+^. The circle represents the universal set containing all possible links *E*_*U*_.

A network can be presented by a simple undirected graph *G* = (*V*, *E*), where *V* is the set of nodes, and *E* is the set of links. Multiple links and self-loops are not allowed. Let *N* = |*V*| and *W* = |*E*| be the number of nodes and number of links, respectively. Let *k*_*i*_ be the degree of node *v*_*i*_, which equals the number of links connected to node *v*_*i*_. We assume that all nodes are known but partial link information is missing. Denote by *E*_*O*_ and *E*_*M*_ the set of observed links and missing links, respectively. Clearly, we have 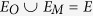. Therefore, the observed network can be presented by *G*_*O*_ = (*V*, *E*_*O*_). We define *α* = |*E*_*M*_|/*W*( ∈ [0,1]) as the proportion of missing link. Denote by *E*_*U*_ = *V* × *V* the universal set containing all *N*(*N* − 1)/2 possible links. The task of link prediction is to reveal the set of missing links *E*_*M*_ from the space of link prediction Ω_*P*_ = *E*_*U*_ − *E*_*O*_. Denote by 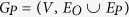 the improved network by adding the predicted links *E*_*P*_(⊆Ω_*P*_). We define the ratio *β* = |*E*_*P*_|/|*E*_*O*_| as the magnitude of additional link information. In general, we have *E*_*P*_ ≠ *E*_*M*_ due to the error predictions. Denote by 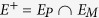 the set of links that are correctly predicted. We use the true positive rate (recall or sensitivity) *R*_*TPR*_ = |*E*^+^|/|*E*_*M*_| to measure the proportion of links that are correctly predicted among the missing links set *E*_*M*_, and the ratio *R*_*PPV*_ = |*E*^+^|/|*E*_*P*_|, i.e., the positive predictive value (precision), to measure the proportion of links that are correctly predicted among the predicted links set *E*_*P*_ . To express the mathematical description of link prediction intuitionally, we give the iceberg diagram for link prediction problem in Fig. 1. In a manner of speaking, the network is like an iceberg. We can only see the part above sea level but do not know the rest under the sea. Link prediction is a technique to infer the invisible part based on the knowledge of observed part.

We identify the targets based on the improved network *G*_*P*_ and then carry out the attack in the original complete network *G*. Note that if a node is attacked, its attached links will be removed together with its removal. Denote by 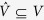 the set of nodes that are attacked (i.e., targets) and 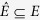 the set of removed links, then the network obtained after node attacks is 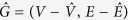. We define the ratio 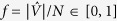 as the strength coefficient of node attacks. Among the many attack strategies^28^ we apply the most used “high degree strategy” in this paper. In this strategy, nodes are attacked according to their rank of degree. i.e., high degree nodes will be attacked firstly. Let 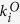 be the degree of node *v*_*i*_ in *G*_*O*_ and 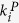 be the degree of node *v*_*i*_ in *G*_*P*_. Without link prediction, we remove nodes in the descending order of the node degree 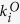. With link prediction, we remove nodes in the descending order of the node degree 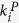. As the attack strength coefficient *f* increases, the network will eventually collapse at a critical value *f*_*c*_ which is generally used to measure the structure robustness of a complex network from the view of defenders. The larger the *f*_*c*_ is, the more robust the network is. Here we employ *f*_*c*_ to evaluate the performance of network disintegration strategy from the view of attackers. Smaller *f*_*c*_ implies more efficient network disintegration.

**Figure 2:**
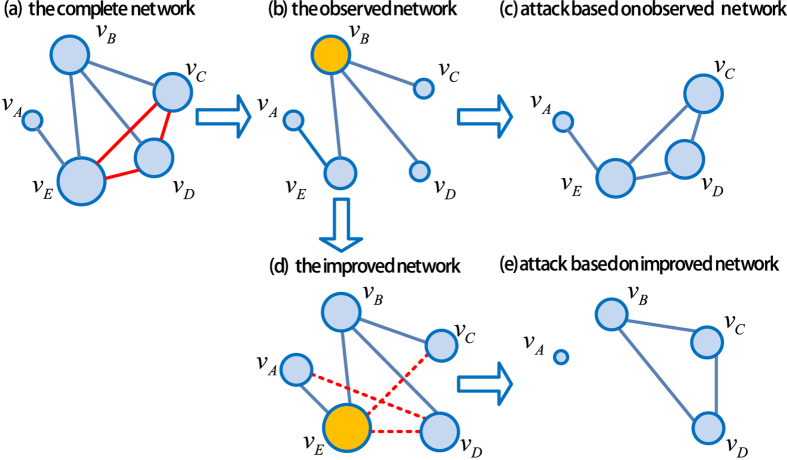
Illustration of network disintegration model based on link prediction. (**a**) The complete network *G*. (**b**) The observed network *G*_*O*_ with three missing links. (**c**) The network obtained after removing the node *v*_*B*_ based on the observed network. (**d**) The improved network *G*_*P*_ with three predicted links added (dotted lines). (**e**) The network obtained after removing the node *v*_*E*_ based on the improved network. The size of each node is proportional to its degree in the current network.

Figure 2 presents a simple example of how our method works. The complete network contains *N* = 5 nodes and *W* = 7 links. The initial degrees of the five nodes in the complete network are *k*_*A*_ = 1, *k*_*B*_ = 3, *k*_*C*_ = 3, *k*_*D*_ = 3, and *k*_*E*_ = 4, respectively. We assume that three links are missing, namely *E*_*M*_ = {*e*_*CD*_, *e*_*CE*_, *e*_*DE*_}. The observed network contains four links, *E*_*O*_ = {*e*_*AE*_, *e*_*BC*_, *e*_*BD*_, *e*_*BE*_}. Then the magnitude of missing link information is *α* = |*E*_*M*_|/*W* = 3/7 and the space of link prediction is Ω_*P*_ = {*e*_*AB*_, *e*_*AC*_, *e*_*AD*_, *e*_*CD*_, *e*_*CE*_, *e*_*DE*_}. Assume we add three links, i.e., *E*_*P*_ = {*e*_*AD*_, *e*_*CE*_, *e*_*DE*_}, predicted by one link prediction algorithm^37^. Then the magnitude of link prediction information is *β* = |*E*_*P*_|/|*E*_*O*_| = 3/4. Among the three links in *E*_*P*_, only *e*_*CE*_ and *e*_*DE*_ are predicted right, i.e., 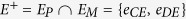. Thus we obtain the sensitivity *R*_*TPR*_ = |*E*^+^|/|*E*_*M*_| = 2/3 and the precision *R*_*PPV*_ = |*E*^+^|/|*E*_*P*_| = 2/3. The degrees of the five nodes in the observed network *G*_*O*_ are 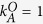, 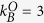, 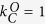, 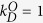 and 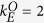, respectively. After the addition of three predicted links, their degrees in the improved network *G*_*P*_ (see Fig. 2(d)) become 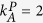, 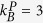, 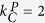, 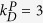 and 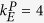, respectively. Based on the observed network *G*_*O*_, the node *v*_*B*_ with the largest degree 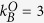 will be removed preferentially as shown in Fig. 2(c), and the network 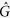 obtained after removing the node *v*_*B*_ is still connected. While based on the improved network, the node *v*_*E*_ with the largest degree 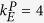 will be removed preferentially as shown in Fig. 2(e), and the network 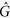 obtained after removing the node *v*_*E*_ is disintegrated into two components.

### Comic effect of link prediction

**Figure 3:**
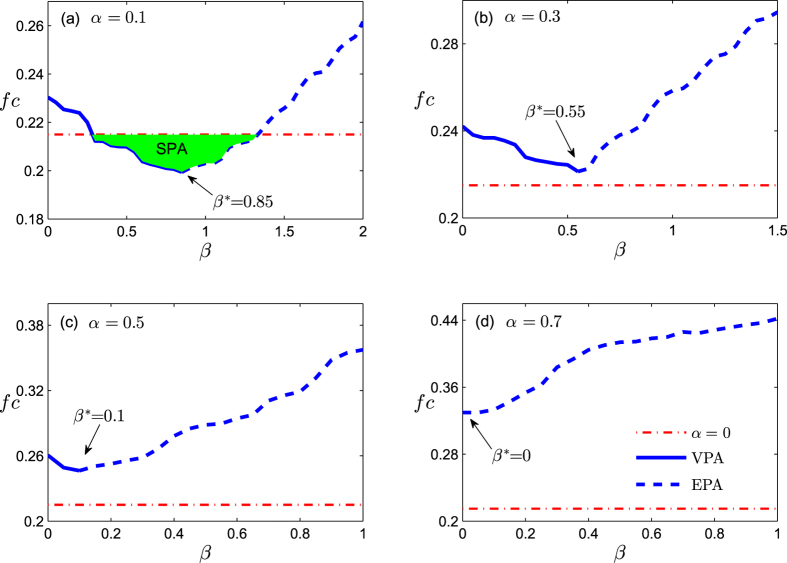
The critical attack strength coefficient *f*_*c*_ versus the magnitude of link prediction information *β* with various magnitude of missing link information *α* in a random scale-free networks. The degree distribution follows *p*(*k*) = (*λ* − 1)*m*^*λ*−1^ *k*^−*λ*^, where *N* = 1000, *λ* = 2.5, and *m* = 2. The results are averaged over 100 independent realizations. The solid lines represent the “valid prediction area” (VPA) and the dash lines represent the “excessive prediction area” (EPA). The dash dotted lines are the reference lines, which represent the case of complete link information, namely *α* = 0. The filled area represents the“surpassing prediction area” (SPA) where *f*_*c*_ is even lower than the case of complete link information.

To analyze the impact of link prediction on network disintergration, we firstly perform experiments on synthetic networks. Due to the ubiquity of scale-free networks with a power-law degree distribution *p*(*k*) ~ *k*^−*λ*^ in real life world, our studies first focus on the network disintegration in scale-free networks. The random scale-free networks with degree distributions *p*(*k*) = (*λ* − 1)*m*^*λ*−1^ *k*^−*λ*^ are generated by using the method proposed in ref. 38. In Fig. 3, we report the dependence of critical attack strength coefficient *f*_*c*_ on the magnitude of link prediction information *β*. We use resource allocation (RA) link prediction algorithm^37^ to predict the missing links. For comparison, we also show the case of complete link information, i.e. *α* = 0, which is usually considered as the ideal case.

**Figure 4:**
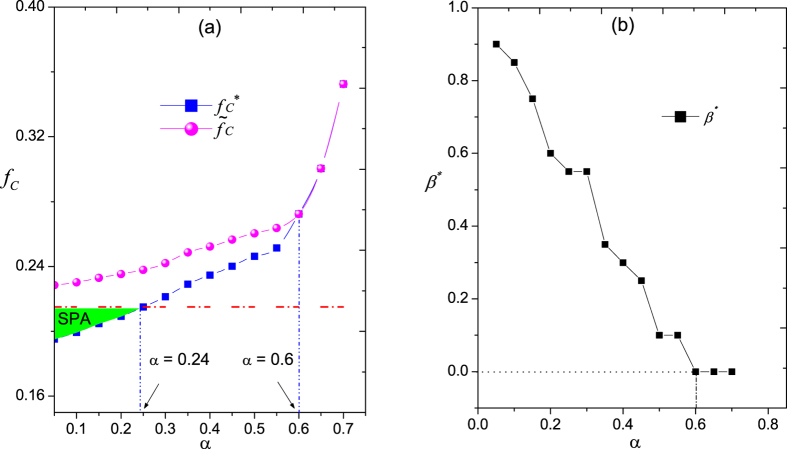
The contribution of link prediction to the network disintegration. (**a**) The optimal critical attack strength coefficient 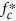 (squares), comparing with the critical attack strength coefficient without link prediction 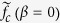 (circles). The horizontal dash dot line presents the value of 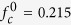 obtained under complete information. (**b**) The optimal magnitude of link prediction information β^*^. The original network is the same as the one we used in Fig. 3. The results are averaged over 100 independent realizations of link prediction.

From Fig. 3, we can see that with the increasing number of missing links, the *f*_*c*_ curve shifts gradually to top-left. For *α* = 0.1, *α* = 0.3 and *α* = 0.5, *f*_*c*_ first decreases with *β* and then increases after *β* > β^*^. We call the region [0,β^*^] the “valid prediction area” (VPA) and the region (β^*^, *β*_*max*_) the “excessive prediction area” (EPA) where the inclusion of any additional predicted links will bring negative effects on the performance of network disintegration. To our surprise, we find an area in which the performance of our method is even better than the “ideal case” where the critical attack strength coefficient is 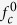. We call the area “surpassing prediction area (SPA)”, see Fig. 3(a). Figure 4(a) shows the performance of network disintegration under the optimal magnitude of link prediction information (i.e., 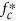), along with the performance of network disintegration without link prediction (i.e., 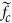 when *β* = 0). The difference between 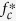 and 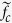 indicates the contribution of the additional links predicted by link prediction algorithm. We find that when *α* < 0.24, 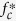 is lower than 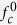, which corresponds to the SPA. It can be explained that the link prediction amplifies the heterogeneity of node importance and reshape the network structure like drawing an exaggerated and characteristic comic. We refer to this phenomenon as the “comic effect” of link prediction. The values of 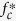 and 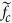 meet at *α* = 0.6, implying that in some cases we can reconstruct the original network to improve the performance of network disintegration even when the network has about 60% links are missing.

It is worth pointing out that, when *α* is large enough, see in Fig. 3(d) when *α* = 0.7, there is no “valid prediction area” and β^*^ = 0. It suggests that link prediction will be counterproductive for the network disintegration performance if overmuch links are missing. The reason is that the link prediction accuracy is usually very low if the prediction based on the observed network with many missing links^39^. These results show that when the link information is not complete, a proper number of additional links can efficiently improve the performance of network disintegration and even obtain better performance (i.e., lower *f*_*c*_) than the case with complete information. It is true that the added links by link prediction may connect to wrong nodes and thus we may not recover the original network completely. However, through link prediction, we partly recover the ranking of node importance, which is really critical in network disintegration.

**Figure 5:**
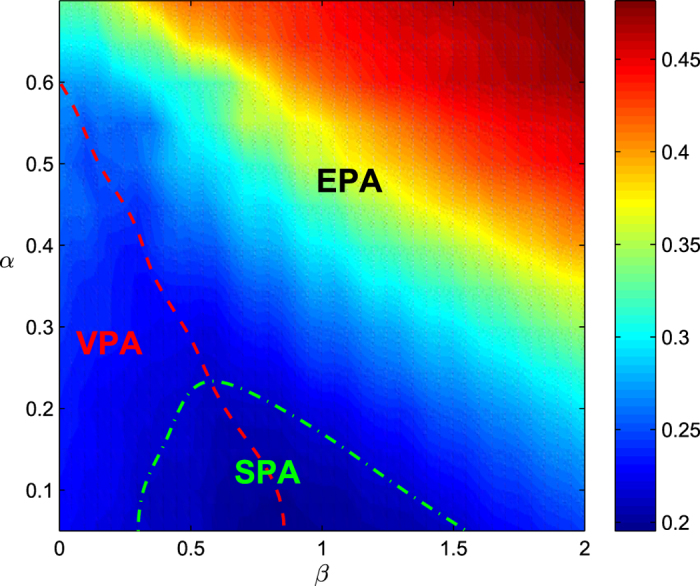
The critical attack strength coefficient *f*_*c*_ in the (*α*, *β*) plane. The original network is the same as the one we used in Fig. 3. The red dash line presents the optimal magnitude of link prediction information β^*^. The left region and the right region of the red dash line are corresponding to the valid prediction area (VPA) and excessive prediction area (EPA), respectively. The area under the green dash dot line is the surpassing prediction area (SPA). The results are averaged over 100 independent realizations of link prediction.

We also show in Fig. 4(b) the optimal magnitude of link prediction information β^*^ as a function of the magnitude of missing link information *α*. We find that β^*^ monotonically decreases with *α* and eventually reaches to zero at about *α* = 0.6, which suggests that the less links are missing, the more predicted links (usually with high accuracy) are required to be added to obtain the best effect. On the contrary, if more links are missing, the less predicted links are added because adding more links will lead to more mistakes due to the low accuracy of link prediction. The dependence of the critical attack strength coefficient *f*_*c*_ on parameter *α* and *β* is shown in Fig. 5, where the VPA, EPA and SPA can be clearly partitioned.

**Figure 6:**
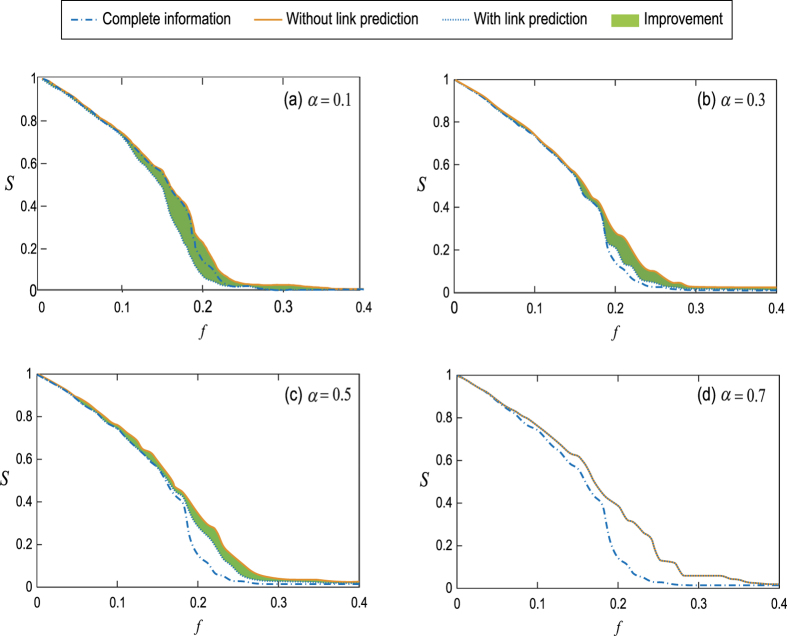
The relative size of giant component *S* versus attack strength coefficient *f* under attacks with complete link information (dash dot lines), attacks without link prediction (dot lines) and attacks with optimal link prediction information (solid lines). The filled area demonstrates the improvement of the effect of network disintegration due to link prediction. The original network is the same network as in Fig. 3. For different *α*, we set *β* = β^*^ as shown in Fig. 3. The results are averaged over 100 independent realizations of link prediction.

The measure *f*_*c*_ is the critical fraction of nodes at which the network completely collapses. However, sometimes we are also interest in the case when the network suffers a big damage without completely collapsing. Figure 6 reports the fraction of nodes in the giant component after node attacks *S* as a function of attack strength coefficient *f* with various magnitude of missing link information *α*. Here we set *β* = β^*^ for corresponding *α*, namely *β* = 0.85 for *α* = 0.1, *β* = 0.55 for *α* = 0.3, *β* = 0.1 for *α* = 0.5 and *β* = 0 for *α* = 0.7. The effect of network disintegration can be characterized by the area under the curve of *S*. The smaller the area is, the more efficient the network disintegration is. Therefore, the area between the curve of *S* with link prediction (dotted lines) and without link prediction (solid lines) demonstrates the improvement of the performance of network disintegration with the assistance of link prediction. The improvement of our method is significant for small *α* and the “comic effect” of link prediction appears in the case of *α* = 0.1, see Fig. 6(a).

### Experiments on real networks

The study of disintegration is important for many real-world systems such as rumor spreading in online social networks, disease transmission through airlines and foodweb. To evaluate the performance of our method, we investigate four real-world networks: (i) the Political blogosphere network (PB)^40^, (ii) the network of the US air transportation system (USAir) (http://toreopsahl.com/datasets/#usairports), (iii) the Foodweb of south Florida during the wet season (Foodweb)^41^ and (iv) the collaboration network between Jazz musicians (Jazz)^42^. Basic statistics of these networks are shown in Table 1. As we can see, all networks are well connected, with high clustering coefficients and short average distances.

**Figure 7:**
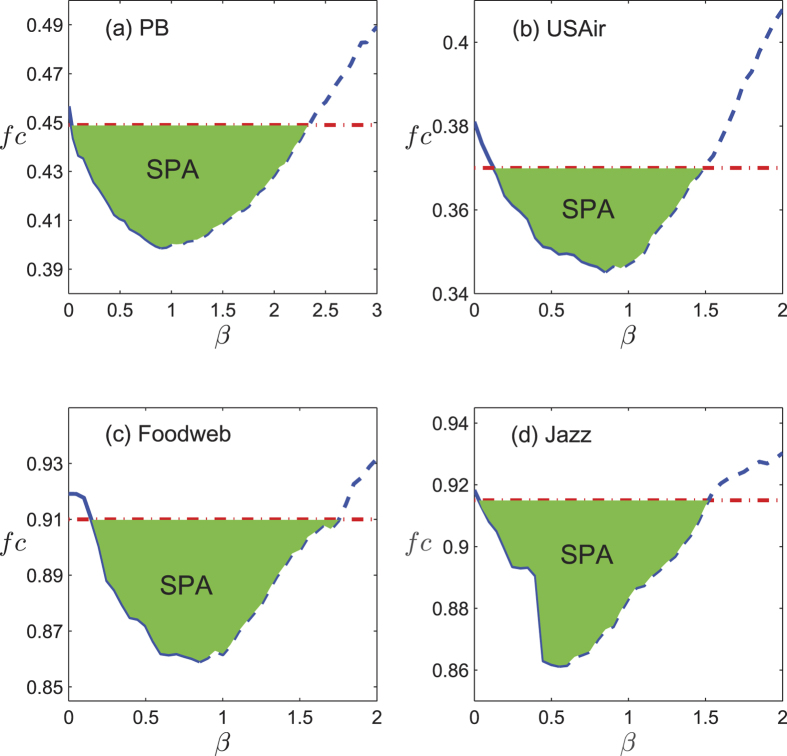
The critical attack strength coefficient *f*_*c*_ versus *β* with a certain missing information accuracy *α* = 0.1 in four real networks. The solid lines represent the “valid prediction area” (VPA) and the dash lines represent the “excessive prediction area” (EPA). The dash dot lines are the reference lines, which represent the case of complete link information, namely *α* = 0. The filled area represents the“surpassing prediction area” (SPA) where *f*_*c*_ is even lower than the case of complete link information. For each *β*, the result is averaged over 100 simulations.

We simulate the prediction and disintegration process on these networks, and results are shown in Fig. 7. All four networks exhibit similar pattern with the synthetic networks: the critical attack strength coefficients, *f*_*c*_ all decrease at the beginning as the ratio of additional links increase, after an optimal ratio, the performance of disintegration degenerates while more links are added. It is interesting to observe that, all the four networks have a large “surpassing prediction area”, where *f*_*c*_ deceases to even below the value obtained under complete information. The SPA for the four networks are *β* ∈ [0.05, 2.35] for PB, *β* ∈ [0.15, 1.45] for USAir, *β* ∈ [0.15, 1.75] for Foodweb and *β* ∈ [0.05, 1.55] for Jazz.

## Discussion

Network disintegration with incomplete link information is an important and challenging problem. In this paper, we introduced the link prediction as a strategy for attackers to improve the performance of network disintegration. We showed that although the missing of link information harms the effect of network disintegration, link prediction can help to improve the performance remarkably. We found with surprise that if the magnitude of missing link information is not too large, the effect of network disintegration with the assistance of link prediction even can be better than the case of complete link information. We called this phenomenon the “comic effect” of link prediction. Although, the link prediction does not recover the missing information completely, but it reshapes the network just like an exaggerated but characteristic comic. As a result, the importance of the key nodes is emphasized by adding a number of predicted links. We believe that the comic effect of link prediction may exist in many backgrounds, not only in the network disintegration. For example, link prediction can not only help to improve the classification accuracy of partially labeled networks^43^ but also be used in recommender systems^44^. These useful applications demonstrate that hidden information revealed by link prediction can help to improve the accuracy of information filtering algorithms.

Moreover, we exposed the area of excessive prediction where the addition of more predicted links will give negative contribution. An optimal magnitude of link prediction information is obtained when the critical attack strength coefficient reaches the minimum. Beyond the optimal magnitude of link prediction information, the contribution of link prediction to the network disintegration will decrease and can even be negative. In addition, we found that the optimal magnitude of link prediction information decreases with the increasing of missing link information, indicating that when there are many missing links it should be very cautious to add new links. For real applications, how to obtain the optimal magnitude of link prediction information for real networks is still an open and challenging problem, as we usually don’t know the portion of missing links and thus it’s difficult to evaluate the algorithm’s performance. According to the results in this paper, by adding a small number of predicted links is usually beneficial when the number of missing links is moderate. Future studies are required to evaluate the choice of appropriate link prediction algorithms to achieve better network disintegration performance^45^.

## Methods

### Algorithms for link prediction

The link prediction problem has been a long-standing challenge in modern information era. Its main goal is to estimate the existence likelihood of nonobserved links based on the known topology and node attributes. The simplest index of link prediction is the common neighbors (CN) index which in common sense, two nodes, *x* and *y*, are more likely to have a link if they have many common neighbors^46^.


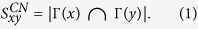


where Γ(*t*) denotes the set of neighbors of node *t*.

Resource Allocation (RA) index^37^ is an improved index based on CN, which assign less-connected neighbors more weight. The index is motivated by the resource allocation dynamics on networks. Consider a pair of nodes, *x* and *y*, which are not directly connected. The node *x* can send some resource to *y*, with their common neighbors being transmitters. The similarity between x and y can be defined as the amount of resource y received from *x*. The mathematical expressions are


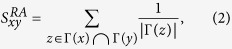


### Performance measurement of network disintegration

In the context of complex networks, the critical removal fraction of nodes *f*_*c*_ for the disintegration of networks is generally used to characterize the network robustness from the view of defenders. The larger *f*_*c*_ is, the more robust the network is. This measure emerged from the random graph theory and was stimulated by Albert *et al.*^4^. Instead of a strict extreme property, it considers statistically how the removal of nodes leads to a deterioration of network performance, and eventually to the collapse of the network at a given critical removal fraction *f*_*c*_. The most common performance measurements include the diameter, the size of the largest component and the average path length. We choose *κ* ≡ 〈*k*^2^〉/〈*k*〉〈2 as the criterion for the collapse of networks^47^,^48^, where the angular brackets 〈.〉 denote an ensemble average. After each node is removed, we calculate *κ*. When *κ* becomes less than 2, we record the number of nodes *t* removed up to that point. The threshold *f*_*c*_ is calculated as *f*_*c*_ = 〈*t*〉/*N*. Here we employ *f*_*c*_ to measure the effect of network disintegration strategy from the view of attackers. Smaller *f*_*c*_ implies more efficient network disintegration.

## Additional Information

**How to cite this article**: Tan, S.-Y. *et al.* Efficient network disintegration under incomplete information: the comic effect of link prediction. *Sci. Rep.* **6**, 22916; doi: 10.1038/srep22916 (2016).

## Figures and Tables

**Table 1:** Basic statistics of four real networks.

Networks	*N*	*W*	〈*k*〉	*C*	*r*	〈*l*〉
PB	1222	16714	27.36	0.36	−0.221	3.65
USAir	1574	28236	35.8	0.384	−0.113	3.14
Foodweb	128	2106	32.906	0.312	−0.111	1.73
Jazz	198	2742	27.7	0.618	0.020	2.23

*N* and *W* are the number of nodes and links. 〈*k*〉 is the average degree; *C* is the clustering coefficient; *r* is the assortativity; 〈*l*〉 is the average shortest distance.
